# Green route to fabrication of Semal-ZnO nanoparticles for efficient solar-driven catalysis of noxious dyes in diverse aquatic environments

**DOI:** 10.3389/fchem.2024.1370667

**Published:** 2024-05-16

**Authors:** Ratan Lal, Tripti Gour, Narendra Dave, Niharika Singh, Jigyasu Yadav, Afshin Khan, Akshita Jain, Lokesh Kumar Agarwal, Yogesh Kumar Sharma, Kuldeep Sharma

**Affiliations:** ^1^ Department of Botany, Mohanlal Sukhadia University, Udaipur, Rajasthan, India; ^2^ Department of Chemistry, Mohanlal Sukhadia University, Udaipur, Rajasthan, India; ^3^ Department of Chemistry, Kalindi College, University of Delhi, Delhi, India

**Keywords:** Semal-ZnO NPs, *Bombax ceiba* L., photocatalytic activity, methylene blue, methyl orange, adsorption kinetics

## Abstract

This work successfully demonstrates a sustainable and environmentally friendly approach for synthesizing Semal-ZnO nanoparticles (NPs) using the aqueous leaf extract of *Bombax ceiba* L. These NPs exhibit an absorption peak at approximately 390 nm in the UV-visible spectrum and an energy gap (E_g_) of 3.11 eV. Detailed analyses of the morphology and particle size using various spectroscopic and microscopic techniques, XRD, FE-SEM with EDS, and HR-TEM reveal crystallographic peaks attributable to the hexagonal phase, with an average crystal size of 17 nm. The Semal-ZnO NPs also exhibit a notable photocatalytic efficiency for degrading methylene blue (MB) and methyl orange (MO) under sunlight in different water samples collected from diverse natural sources, indicating that they are promising photocatalysts for environmental remediation. The photocatalytic efficiency of the biofabricated Semal-ZnO NPs is impressive, exhibiting a photodegradation rate of up to 99% for MB and 79% for MO in different water samples under exposure to sunlight. The novel phytofabricated Semal-ZnO NPs are thus a beacon of hope for the environment, with their desirable photocatalytic efficiency, pseudo-first-order kinetics, and ability to break down noxious dye pollutants in various aquatic environments.

## Introduction

Industrialization and technological progress are considered the backbone of development of any nation, but they are equally responsible for the accumulation of life-threatening wastes in water (Marimuthu et al., 2020; Amdeha and Mohamed, 2021; Lemessa et al., 2023; Manna and Sen, 2023). Water is one of the most precious natural resources that should not be challenged by human industrialization and everyday use as a commodity (Chelliah et al., 2023). Massive population growth, exponentially increasing industrial processes, and innovative developments have adversely affected the global water quality (Xiao et al., 2023). Industrial processes involving the manufacture of cosmetics, textiles, leather, paper, plastic, and dyes often result in the discharge of wastewater contaminated with complex and vividly colored dyes, which ultimately leads to contamination of water resources and reservoirs (Nagajyothi et al., 2020; Alamier et al., 2023; Khader et al., 2023; Peng et al., 2023). These dye molecules are aromatic, chemically stable, toxic, potentially mutagenic, and carcinogenic, in addition to being typically persistent and resistant to natural degradation processes (Suhaimi et al., 2022; Aslam et al., 2023; El Sharkawy et al., 2023; Geldasa et al., 2023; Vievard et al., 2023). Their presence in water bodies has substantial detrimental effects on aquatic life and the overall ecosystem, posing significant environmental challenges and threats to human life if left untreated (Alfei et al., 2023; Warren-Vega et al., 2023). Therefore, dye degradation is a pivotal component of wastewater treatment in terms of sustainable removal of the dye pollutants contaminating industrial effluents and is essential for safeguarding the quality of water resources.

Traditional treatment methods involving coagulation, adsorption, and osmotic pressure have been used to remove dyes from rivers and other water bodies; however, each method has its limitations in efficiently eliminating dyes from wastewater, necessitating advanced and efficient degradation techniques (Ekennia et al., 2021; Zewde and Geremew, 2022). Innovative catalytic photodegradation has emerged as a promising solution to address the challenges posed by wastewater contaminated with organic dyes (Mutukwa et al., 2022; El Sharkawy et al., 2023). Catalytic photodegradation is easy to achieve, is affordable, and has a straightforward instrumental procedure as well as non-selective oxidation. Moreover, it ensures complete degradation of the organic dyes into simpler and less harmful substances, making the wastewater suitable for discharge or further treatment (Khan et al., 2023; Motelica et al., 2023).

Catalytic photodegradation harnesses the power of catalysts, which are usually semiconductor materials like titanium dioxide (TiO_2_) or zinc oxide (ZnO), in combination with light energy to initiate breakdown of the dye molecules (Geldasa et al., 2023; Kaushik et al., 2023; Kumari et al., 2023). This approach offers several advantages, including high efficiency, reduced chemical usage, and the potential for treating a wide range of dye types by breaking them down into simpler, less harmful substances (Marimuthu et al., 2020; Khan et al., 2023). A photocatalyst, upon exposure to light, generates reactive oxygen species (ROS) like hydroxyl radicals that initiate the degradation reactions (Sarkar et al., 2020). Both visible and UV radiation may be utilized to create and excite the electron–hole pairs necessary for breakdown of the impurities depending on the bandgap energies of the semiconductors used (Ahmad and Kalra, 2020; Gadore et al., 2023). In this context, the synthesis of biogenic nanoparticles (NPs) for photodegradation is a groundbreaking yet ecofriendly solution for addressing the challenges of environmental remediation and sustainable water resource management (Kumari et al., 2023; Salmi et al., 2023; Yadav et al., 2023). Traditional approaches to nanoparticle syntheses often involve chemical and physical methods that may introduce toxic reagents and generate hazardous byproducts (Masood et al., 2023). In recent years, there has been a growing interest in the synthesis of NPs using biogenic methods by harnessing the potential of biological entities, such as microorganisms, plants, and algae (Sharma et al., 2016; Al-Askar et al., 2023; Supin et al., 2023). By leveraging the reducing and stabilizing abilities of the biomolecules, such as the enzymes, proteins, and biochemicals, of such biological entities, biogenic NPs have been synthesized that are not only environmentally benign but also cost-effective and easily scalable (Alprol et al., 2023; Chamaraja et al., 2023; Fouda et al., 2023). The photocatalytic activities of the biogenic NPs are attributed to their specific surface chemistry and morphologies, which facilitates generation of ROS upon exposure to light (Gul et al., 2023). The unique properties of these biogenic NPs offer numerous advantages, such as biocompatibility, reduced toxicity, and enhanced catalytic efficacy, making them well suited for catalytic photodegradation of harmful dyes (Aaga and Anshebo, 2023; Malik et al., 2023; Meena et al., 2023). In the present study, an aqueous leaf extract of the Semal tree was used to synthesize zinc oxide (Semal-ZnO) NPs in an environmentally friendly manner. The cosmopolitan Semal tree *Bombax ceiba* is a member of the Malvaceae family and has great medicinal and ethnobotanical significance as its different plant parts like root, stem, leaves, flowers, and seeds possess various bioactive substances, such as lupeol, β-sitosterol, shamimicin, ceibanaphthoquinone, simalin-A, simalin-B, mangiferin, epicatechin-3-O-b-xylopyranoside, epicatechin-7-O-b-xylopyranoside, shamiminol, stigmasta-3,5-diene, lupenone, opuntiol, quercetin, shamimin, palmitic acid, and polysaccharides (Chauhan et al., 2018; Khurshid Alam et al., 2018; Nikita and Shweta, 2020; Shukla et al., 2020).

Therefore, the present work explores a green synthesis method for biogenic NPs by emphasizing their eco-friendly nature and potential for large-scale production. The structural and morphological properties were also investigated to understand their suitability and modes of action for catalytic photodegradation applications. Furthermore, their performance and efficacy for degrading dye pollutants in natural freshwater samples as well as potential for integration into wastewater treatment systems under environmentally benign and ambient conditions were explored.

## Materials and methods

### Materials

The green leaves of *B. ceiba* (Semal) were obtained locally from the botanical garden of the Department of Botany, University College of Science, Mohanlal Sukhadia University, Udaipur, Rajasthan, India. Zinc nitrate hexahydrate [Zn(NO_3_)∙6H_2_O], methylene blue (MB; molecular weight = 319.85 g/mol), and methyl orange (MO; molecular weight = 327.33 g/mol) were procured from Sigma-Aldrich and Merck. Deionized water (CDH, India) was used throughout the reaction process.

### Methods

The green leaves of *B. ceiba* (Semal) collected locally were carefully washed with running tap water and distilled water to remove dust and dirt, followed by drying for 2 weeks at room temperature (RT) in a dust-free environment to reduce the moisture content before finally being homogenized into a fine powder. The aqueous pale-yellow solution was filtered using Whatman filter paper No. 1, and the resulting filtrate was either utilized immediately for NP synthesis or maintained in storage at 4°C for future use.

### Synthesis of Semal-ZnO NPs

The NP production process involved combining 1 g of zinc nitrate hexahydrate with 25 mL of the preheated pale-yellow aqueous solution (pH 10), and the mixture was agitated and heated up to 60°C using a magnetic stirrer heater until it transformed into a suspension with a vivid yellow tint. The suspension was carefully poured into a ceramic crucible and annealed in a muffle furnace at 400°C for 2 h. The annealing process was carefully monitored until a white crystalline powder was obtained. This powder was then expertly ground using a mortar and pestle and stored in high-quality Borosil^®^ glass vials for the necessary physicochemical characterizations.

### Characterizations of Semal-ZnO NPs

The optical absorption spectrum of the ZnO NPs was obtained in the scanning range of 300–600 nm using a double-beam UV-visible spectrophotometer (Shimadzu 1900i, Japan). The powder X-ray diffraction (PXRD) pattern was then obtained using a powder diffractometer (X-ray diffractometer Ultima IV, Rigaku, Japan) with Cu-Kα radiation (λ = 0.154060 nm) in the 2θ range from 200° to 800° to determine the crystallinity, particle size, and purity of the biogenic NPs. Electron micrographs were obtained via high-resolution transmission electron microscopy (HR-TEM) for sample size >200 as well as field-emission scanning electron microscopy (FE-SEM) at 200 kV (JEOL JEM-2100F, Japan) with energy dispersive X-ray spectroscopy (EDS) at 30 kV (JEOL JSM-7600F, Japan) and examined using ImageJ software to determine the morphological features and elemental constituents. To provide the molecular composition of the biosynthesized material and elucidate the role of the biomolecule in the formation of NPs, the aqueous extract and phytofabricated samples were subjected to Fourier-transform infrared (FT-IR) spectroscopy.

### Photocatalytic activity

The photocatalytic activity of the NPs was assessed using two noxious dyes, namely, MB (pH 8.0) and MO (pH 4.0), under solar irradiation in April and May. The photodegradation of the dyes was carried out as follows: initially, each dye was dissolved in deionized water, and the aqueous dye solution (10 mg/L) to be decomposed was taken along with 25 mg of the catalyst (ZnO NPs) in a glass conical flask of volume 50 mL (Borosil^®^) and constantly stirred in the dark at RT for 30 min to achieve the adsorption–desorption equilibrium before irradiation. Aliquots (4.0 mL each) were pipetted out periodically from the reaction mixture at 10-min intervals. The periodic measurements of MB and MO concentrations were performed at different wavelengths of 664 and 463 nm, respectively, using a UV-vis spectrophotometer (Shimadzu 1900i, Japan).

The photocatalytic degradation performance was defined using the following equation:

$$\text{Degradation }\left(\%\right)=\left(\text{Co }–\text{ Ct}\right)/\text{Co}*100,$$

where Co is the initial concentration (t = 0) and Ct is the final concentration of the dye in the solution at a given time (t = t min).

The following kinetic equation was used to further analyze the results:

$$\text{kt}=\ln\left(\text{Co}/\text{Ct}\right),$$

where Co and Ct are the respective dye concentrations before and after irradiation, k is the rate constant, and t is the reaction time consumed during photodegradation of the organic dye.

### Efficiency of Semal-ZnO NPs for photodegradation in natural water samples

The photocatalytic efficiency of the Semal-ZnO NPs was determined using two noxious dyes, MB (pH 8.0) and MO (pH 4.0), dissolved in different water samples and exposure to sunlight. The water samples were collected from different natural freshwater sources, namely, Fateh Sager (FS), Pichola Lake (PL), Ayad River (AD), borewell (BL), and tap water (TP), in the City of Lakes, Udaipur, India, at different time intervals in 250-mL glass reagent bottles (Borosil^®^). These water samples collected from different sources were filtered using Whatman filter paper No. 1, pooled, and stored separately at 4.0°C for further analyses.

## Results and discussion

### Optical studies

**FIGURE 1:**
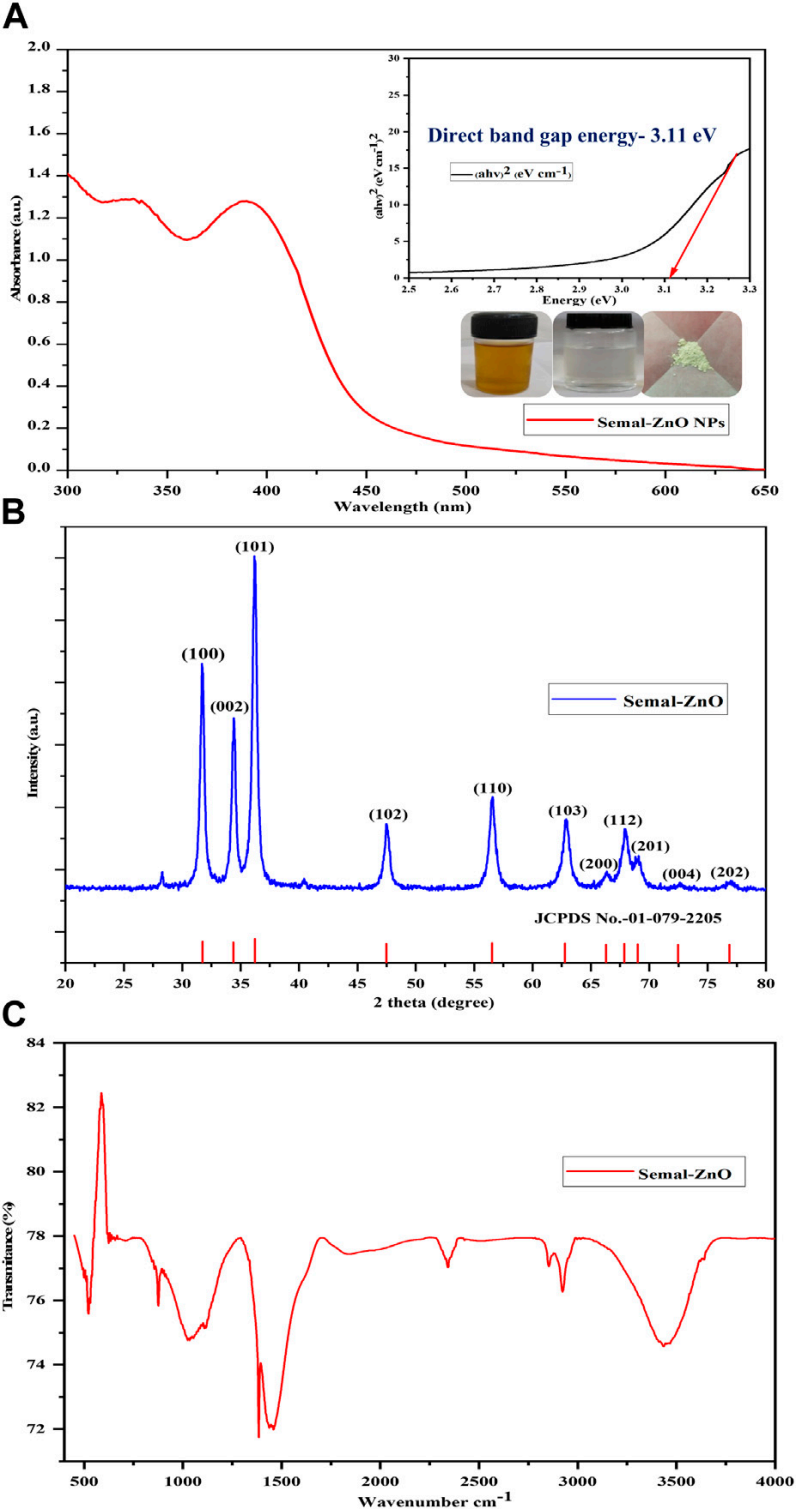
**(A)** UV-vis absorbance spectrum and Tauc plot, **(B)** PXRD pattern, and **(C)** FT-IR spectrum of the biofabricated Semal-ZnO NPs.

The UV-visible spectrophotometer was used to monitor the formation of the green synthesized Semal-ZnO NPs in the range of 300–600 nm. The maximum absorption depends on the NP morphology, dimensions, and surface microstructure. A broad absorption peak centered at approximately 390 nm was the characteristic of the Semal-ZnO NP formation in the range of 350–400 nm and was attributed to the binding of various capping molecules with the ZnO NPs, resulting in increased bandgap (Figure 1A) (Abdelbaky et al., 2022; Sharma et al., 2022; Ramesh et al., 2023). The absorption peak observed in this study may be attributed to the intrinsic bandgap formed by electron transitions from the valence band (E_V_) to the conduction band (E_C_) of the Semal-ZnO NPs (Mageswari et al., 2023). This peak confirms the efficient synthesis of Semal-ZnO NPs from *B. ceiba* (Semal) leaf extract. The Tauc plot was used to determine the optical bandgap energy (E_g_) as 3.11 eV (Figure 1A) for the Semal-ZnO NPs. The bandgap value (E_g_) for the green synthesized Semal-ZnO NPs was in good agreement with the values of 2.88 and 3.10 eV (Ekennia et al., 2021); 3.10, 3.12, and 3.07 eV (Almarhoon et al., 2022); and 3.26 and 3.3 eV (Alharthi et al., 2020) noted in literature using the equations E_g_ = (hc/ʎ)*eV and E_g_ = (1240/ ʎ)*eV, where E_g_ is the bandgap energy (eV), h is Planck’s constant (6.626 × 10^–34^ Js), c is the velocity of light in vacuum (3 × 10^8^ m/s), and λ is the wavelength (nm) (Tauc et al., 1966).

### PXRD patterns

The crystalline phase of the green synthesized Semal-ZnO NPs from *B*. *ceiba* leaf extract was characterized by PXRD analysis. All the observed diffraction peaks were well indexed with the hexagonal phase (wurtzite structure) of Semal-ZnO when compared to the standard values of ZnO (JCPDS no. 1-079-2205), which was used as the reference with lattice parameters a = 3.2501 Å and c = 5.2071 Å using X’Pert HighScore Plus software (Figure 1B) (Abdelbaky et al., 2022; Chandrasekaran et al., 2023). The diffraction pattern revealed that all samples were in the same position and that no additional peaks of any alternative species were found, proving that all of the precursors were completely decomposed during the process (Figure 1B) (Sreelekshmi et al., 2023). The results also demonstrated that diffraction peaks corresponding to impurities were absent in the PXRD patterns, confirming the high purity of the synthesized Semal-ZnO NPs (Meena et al., 2023; Mishra et al., 2023). For rearrangement of the atomic grouping, high heat treatment would probably supply enough kinetic energy for formation of the NP crystal structure. Furthermore, this method produces Semal-ZnO at a sufficiently high temperature of approximately 400°C, resulting in a crystalline structure (Eissa et al., 2022). The sharp and narrow peaks of the biosynthesized Semal-ZnO NPs appear to have a high degree of crystallization (Shochah and Jabir, 2023). The crystallite sizes and degree of crystallinity of the Semal-ZnO NPs were assessed through the Debye–Scherrer formula.

D=0.94λ/β⁡cos⁡θ,

where λ is the wavelength of the X-ray, β is the full-width at half maximum (FWHM) in radians, and θ is the diffraction angle. Some other parameters, such as the d-spacing, area under the main peaks, and FWHM of these peaks, were also analyzed. The results show that the crystallite size of the green synthesized Semal-ZnO NPs is in the range of 5–40 nm.

### Functional group analysis

The FT-IR spectra were recorded for both the aqueous Semal leaf extract and a representative sample of the Semal-ZnO NPs in the infrared scanning range from 4,000 to 400 cm^-1^ at RT using KBr pellets to evaluate the functional groups of the plant metabolites involved in the reduction and stabilization of the Semal-ZnO NPs (Figure 1C) (Ekennia et al., 2021; Supin et al., 2023). The peaks observed at 3,855 cm^-1^ to 3,017 cm^-1^ were interpreted as the sharp stretching vibrations of the O-H group in water, alcohol, and phenol present in the Semal leaf extract (Abdullah et al., 2023; Elbrolesy et al., 2023). The O=C=O strong stretching of carboxylic acid was observed at 2392 cm^-1^ and 2328 cm^-1^; the C=C medium stretching of the alkane groups was attributed to the band at 1,460 cm^-1^ (Meena et al., 2023), and the bands at 1,382 cm^-1^ and 874 cm^-1^ were associated with C-O strong stretching in the aliphatic ether compounds and C-H strong banding in the 1,3-disubstituted compounds, respectively (Vinayagam et al., 2020). The strong sharp peak at 522 cm^-1^ accompanying the Zn-O stretching vibrations in the spectrum verified the formation of the Semal-ZnO NPs (Lanjwani et al., 2023; Supin et al., 2023). The FT-IR results suggest that the range of functional groups (alcohols, phenols, ether, amines, and aromatic and aliphatic amines) present in the aqueous Semal leaf extract is likely associated with different phytochemicals, such as alkaloids, saponins, tannins, phenols, terpenoids, triterpenoids, flavonoids, and steroids. These phytochemicals would have likely played dominant roles in the green synthesis process and probably participated in the reduction and stabilization process during formation of the NPs in the aqueous medium.

### FE-SEM analysis

**FIGURE 2:**
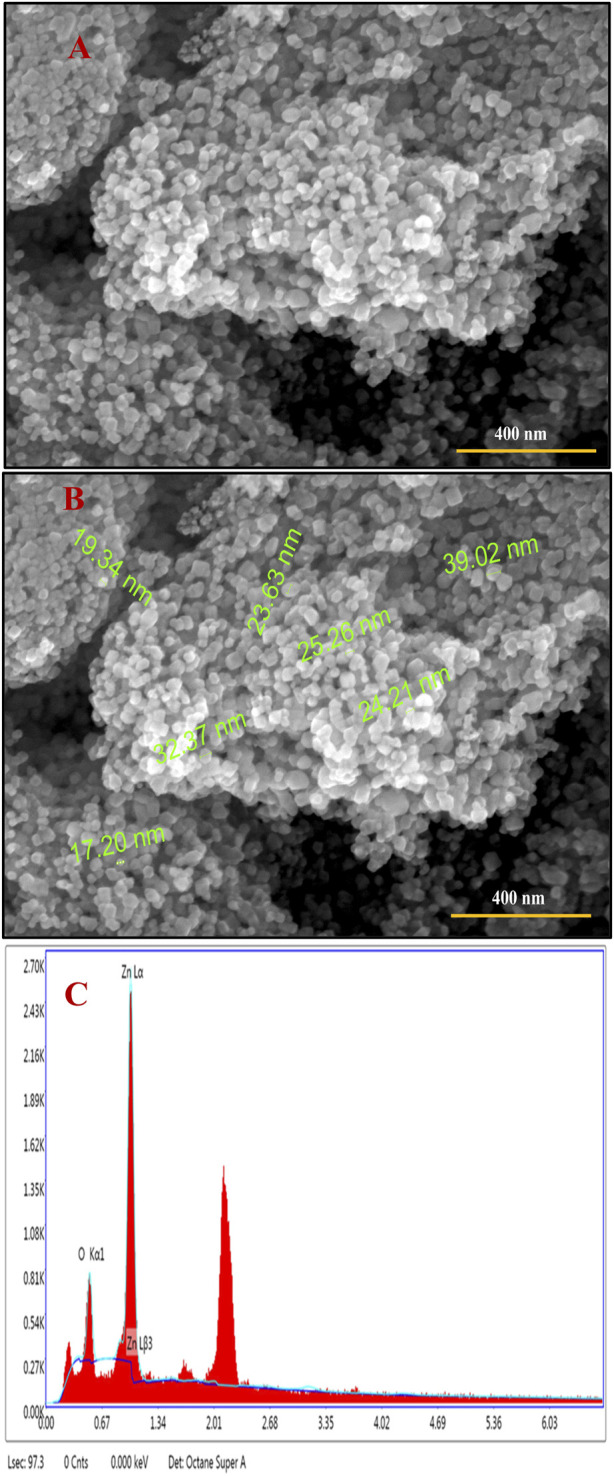
**(A, B)** FE-SEM images and **(C)** EDS spectrum showing the elemental constituents of the biofabricated Semal-ZnO NPs.

FE-SEM is a high-resolution surface imaging technique that provides insights into nanostructures at the microscopic level (Hussein and Mohammed, 2021; Abdelbaky et al., 2022). This technique employs an electron beam to capture surface images with higher magnification and larger field depth, providing surface topological information of various nano-objects depending on the electron density of the surface (Saif et al., 2019; Pillai et al., 2020). Figures 2A,B show the surface morphologies of the Semal-ZnO NPs under different magnifications; the SEM images show that most of the Semal-ZnO NPs are spherical and have a synthesized diameter range of 5–35 nm. The green Semal-ZnO NPs are also found to be aggregated and homogeneous owing to the stacking morphology (Abdelbaky et al., 2022; Purkait et al., 2023; Sreelekshmi et al., 2023; Supin et al., 2023). The agglomerated particles indicate larger sizes with low surface-to-volume ratios. The size and shape of the NPs depend upon the nature of the capping agent present in the aqueous extract of Semal leaves (Almarhoon et al., 2022). The SEM-EDS examinations confirmed the purity and elemental composition of the Semal-ZnO NPs synthesized from the aqueous leaf extract of *B. ceiba.* The SEM-EDS micrographs show the presence of zinc and oxygen elements in the spectrum, with strong signal energy peaks for Zn and weak signal energy peaks for O in the atomic percentage of 66.93% and 33.07%, respectively. No additional peaks were observed in the micrographs, indicating the high purity of the Semal-ZnO NPs synthesis.

### HR-TEM analysis

**FIGURE 3:**
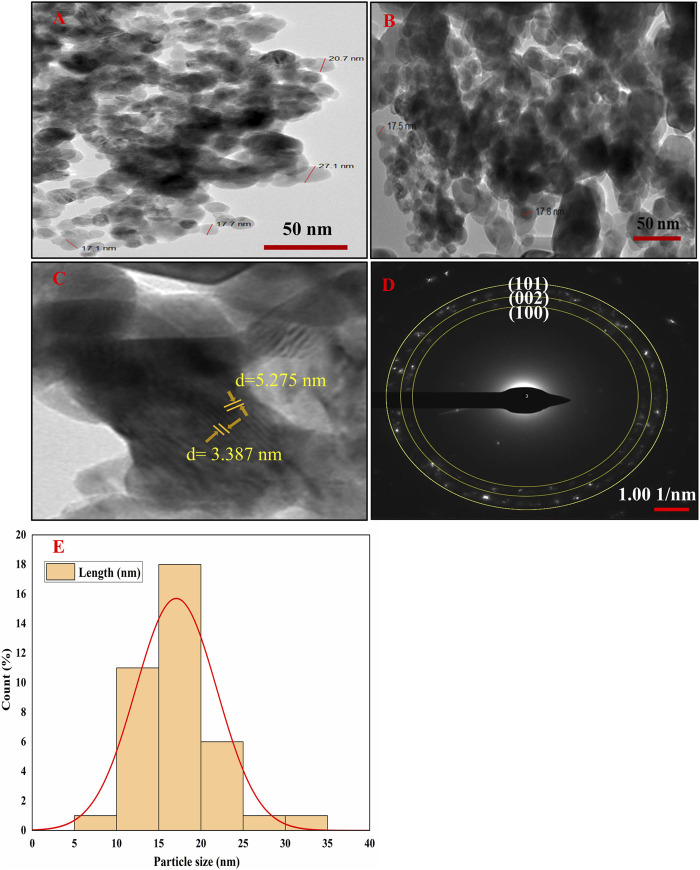
**(A–D)** HR-TEM images and **(E)** particle size distribution histogram of the biofabricated Semal-ZnO NPs.

HR-TEM was used to ascertain the sizes, shapes, and crystalline makeup of the phytofabricated NPs, and the resulting electron micrographs exhibit that the Semal-ZnO NPs are spherical with an average size of 17 nm (Figures 3A,B,E), which is in line with the findings of some recent literature (Kamaraj et al., 2022; Sreelekshmi et al., 2023; Tejaswini et al., 2023; Yassin et al., 2023). The micrographs show a homogeneous distribution, but agglomeration of the Semal-ZnO NPs was also visible (Abdullah et al., 2023; Meena et al., 2023), probably owing to the elevated surface energy of the NPs and chemical makeup of the surface-bound capping and reducing agents (Abdullah et al., 2023). Furthermore, there is a propensity for the NPs to attract each other and form larger clusters in an aqueous medium, thereby altering the properties of the synthesized materials (Alprol et al., 2023). Fringes were observed in the TEM selected area electron diffraction (SAED) pattern at distances of 3.0125, 2.7129, and 2.4157, respectively, corresponding to the (100), (002), and (101) hkl planes (Baruah et al., 2021) (Figures 3C,D), indicating the crystalline nature of the NPs. This is strongly corroborated by the XRD results, which demonstrates that the particles were crystalline (Rambabu et al., 2021; Shobha et al., 2023).

### Photocatalytic activity

**FIGURE 4:**
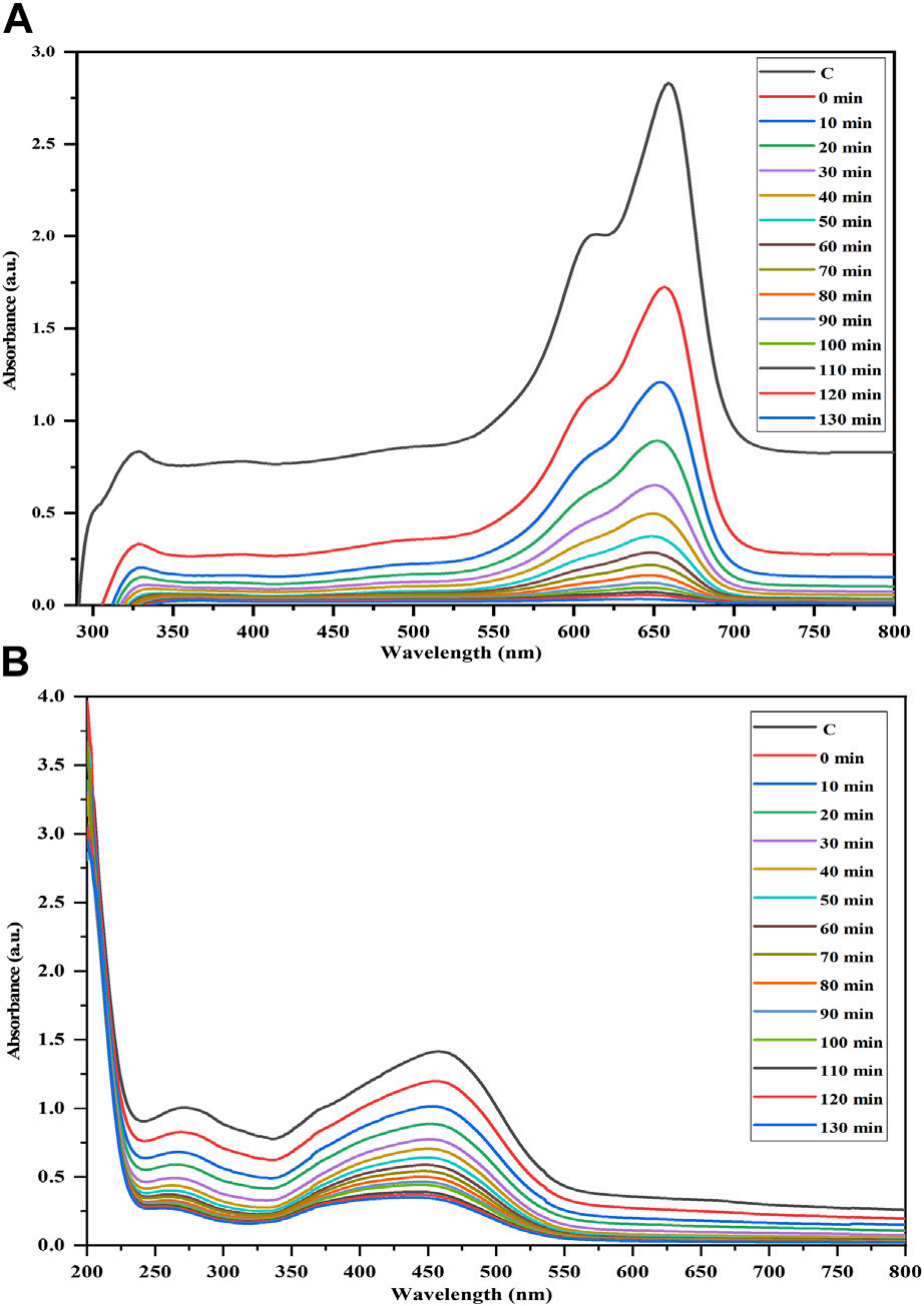
Time-dependent UV-vis absorption spectra of the photocatalytic degradations of **(A)** MB and **(B)** MO dyes in the presence of the biofabricated Semal-ZnO NPs.

**FIGURE 5:**
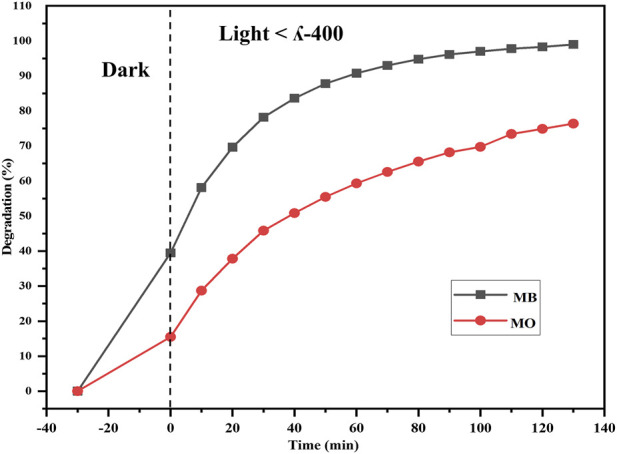
Degradations (%) of the MB and MO dyes using the biofabricated Semal-ZnO NPs.

The efficacy of the Semal-ZnO NPs was assessed in terms of their photocatalytic performance via degradation of MB and MO over different time intervals under ambient conditions, thus providing insights into their potential application in wastewater treatment. Experiments were performed by adding the Semal-ZnO NPs to water samples containing these two dyes, and the hues of the solutions changed significantly from blue and orange to colorless for MB and MO, respectively. Periodic measurements of the MB and MO concentrations were performed using UV-vis spectroscopy at wavelengths of 664 and 463 nm, respectively, and the absorption peaks decreased steadily with increasing exposure time, indicating the photocatalytic degradation of both MB and MO (Figures 4A,B). The total degradation percentages of MB and MO using Semal-ZnO NPs was found to be 99% and 79%, respectively, after 130 min of exposure to sunlight (Figure 5). The phytofabricated Semal-ZnO NPs exhibited a remarkably high capacity for MB degradation in the presence of sunlight than MO dye.

The organic dye molecules are initially adsorbed onto the active sites of the Semal-ZnO NPs due to their high activity and specific surface area, following the principles of heterogeneous reactions. When the Semal-ZnO NPs are exposed to solar energy higher than their bandgap, holes are created in the valance band (h VB^+^) and electrons are created in the conduction band (e^−^ CB^–^). These photogenerated electron–hole pairs are essential for the breakdown of the dye molecules. The excited dye molecules from solar radiation can also introduce electrons into the conduction band of the ZnO NPs.

$$\text{Semal}-\text{ZnO}+\text{hv }\to \text{Semal}-\text{ZnO }\left(e^{-}{\text{CB}}^{-}+h\;{\text{VB}}^{+}\right)$$

$$\text{Dyes}+\text{hv }\to {\text{Dyes}}^{*}\;\left(\text{MB},\text{MO}\right)$$

$$\text{Dyes}+\text{Semal}-\text{ZnO }\to \text{Dyes}+\text{ZnO }\left(e^{-}\;{\text{CB}}^{-}\right)$$

The h VB^+^ reacts with H_2_O or OH^−^ to form hydroxyl radicals (HO•) or can even directly oxidize the dye molecules to form degradation products.

$$\text{Semal}-\text{ZnO }\left(h\;{\text{VB}}^{+}\right)+H_{2}O\;\to \text{Dye}+\text{Semal}-\text{ZnO}+H^{+}+{\text{HO}}^{∙}$$

$$\text{Semal}-\text{ZnO }\left(h\;{\text{VB}}^{+}\right)+{\text{OH}}^{-}\;\to \text{ Semal}-\text{ZnO}+{\text{HO}}^{.}$$

$$\text{Semal}-\text{ZnO }\left(h\;{\text{VB}}^{+}\right)+\text{Dye products }\to \text{Degradation of dyes}$$

While following the necessary sequence of events, (e^**-**^ CB^-^) reacts with the O_2_ molecules adsorbed on the surface of the ZnO NPs to create HO..

$$\text{Semal}-\text{ZnO }\left(e^{-}\;{\text{CB}}^{-}\right)+O_{2}\;\to \text{Semal}-\text{ZnO}+O_{2}^{.-}$$

$$O_{2}^{.-}+H^{+}\;\to \;{\text{HO}}_{2}^{.}$$

$$2{\text{HO}}_{2}^{.}{\;\to H}_{2}O_{2}+O_{2}$$

$$H_{2}O_{2}+O_{2}^{.}{\;\to \text{HO}}^{.}+{\text{OH}}^{-}+O_{2}$$

$$H_{2}O_{2}+\text{Semal}-\text{ZnO }\left(e^{-}\;{\text{CB}}^{-}\right)\;\to \text{Semal}-\text{ZnO}+{\text{HO}}^{.}+{\text{OH}}^{-}$$

$$H_{2}O_{2}+\text{hv }\to 2{\text{HO}}^{.}$$

The hydroxyl radicals produced are very powerful oxidizers, and they react with the dye molecules along with other oxidant species such O_2_•^-^ and HO_2_^**.**^ to produce the degradation products (Alharthi et al., 2020; Golmohammadi et al., 2020; Aldeen et al., 2022; Albo Hay Allah and Alshamsi, 2023; Meena et al., 2023).

$$\text{MB}*/{\text{MO}}^{*}+{\text{HO}}^{.}\;\to \text{Degradation of organic pollutants}$$

$${\text{MB}}^{*}/{\text{MO}}^{*}+O_{2}^{.}-\;\to \text{ Degradation of organic pollutants}$$

$${\text{MB}}^{*}/{\text{MO}}^{*}+{\text{HO}}_{2}^{.}\;\to \text{Degradation of organic pollutants}$$

The eco-friendly synthesis of ZnO NPs from the aqueous leaf extract of Semal in this study reduces the chemically induced toxicity while increasing the dye degradation efficiency, which is consistent with the findings of previous studies that have shown the strong photocatalytic activities of biologically synthesized ZnO NPs (Nguyen-Hong et al., 2021; Rambabu et al., 2021; Wongrerkdee et al., 2023).

### Adsorption kinetics

Adsorption kinetics in the context of dye degradation is crucial for environmental remediation and wastewater treatment as it indicates how the dyes are removed or degraded from industrial effluents or stagnant water bodies by adsorption onto solid surfaces; this is because dyes can be harmful to the environment and human health if released into water channels and aquifers (Khan et al., 2021; Haleem et al., 2023). Adsorption kinetics studies help to determine a suitable adsorbent material, predict the required contact time, and optimize the operating conditions to achieve efficient dye degradation (Ghaffar et al., 2023; Usman et al., 2023). The kinetics of dye degradation through adsorption involves understanding the rates at which dye molecules are adsorbed onto the adsorbent materials and can be described using various mathematical models, with the most commonly used types being the pseudo-first-order and pseudo-second-order kinetics (Borah et al., 2023; Jaramillo-Fierro et al., 2023; Singh et al., 2023; Usman et al., 2023).

The present study focuses on the photocatalytic degradation of two dyes, MB and MO, under ambient conditions via graphs plotted using the ratio of dye concentration C to initial dye concentration Co (C/Co) at various durations of exposure to sunlight.

The following kinetic equation was used to further analyze the results:

$$\text{kt}=\ln\left(\text{Co}/\text{Ct}\right)\ldots \ldots \ldots \ldots \ldots \ldots .\;\left(\text{Pseudo }-\text{first}\;\text{order kinetics equation}\right),$$

where Co and Ct are the dye concentrations before and after irradiation, k is the rate constant, and t is the reaction time consumed during photodegradation of the organic dye (Ebrahimian et al., 2021; Lanjwani et al., 2023).

**FIGURE 6:**
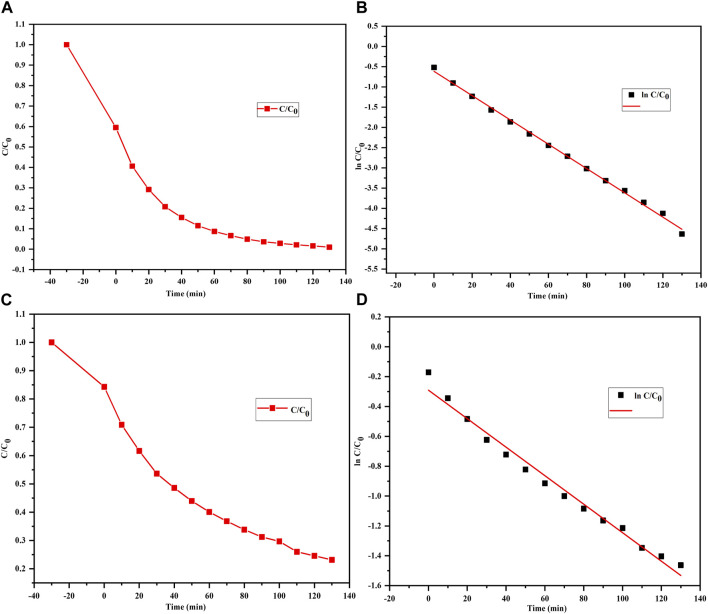
Photodegradation curves of **(A, B)** MB and **(C, D)** MO dyes treated with the biofabricated Semal-ZnO NPs showing pseudo-first-order kinetics.

The plots of ln(Co/C) against time (Figures 6A–D) allow visualization of how the concentrations of the MB and MO dyes change over time during the photodegradation process, and the photodegradation of both MB and MO is seen to follow a pseudo-first-order kinetic model (Asif et al., 2023). This means that the rates of degradation of MB and MO are directly proportional to the remaining concentrations of the dyes, and the natural logarithm of the ratio (Co/C_t_) exhibits a linear relationship with time and is in line with the findings of other reports (Farouq, 2022; Singh et al., 2023).

### Adsorption mechanism

With respect to the common mechanisms of adsorption of charged molecules like dyes, the surface charges and porosities of the particles, functional groups present on the adsorbents, and types of adsorbate have significant impacts on the effectiveness of adsorption (Amdeha, 2023; Kumari et al., 2023). Cationic and anionic dyes have different charges and are thus attracted differentially to the charged surface of an adsorbent, which can lead to different interactions like electrostatic interactions and hydrogen bonding with the surface of the adsorbent (Tan et al., 2015; Chauhan et al., 2020; Nguyen-Hong et al., 2021). Cationic (positively charged) dyes may experience attractive electrostatic interactions with the negatively charged functional groups on the adsorbent surface (e.g., O-H groups); similarly, anionic (negatively charged) dyes may experience attractive electrostatic interactions and hydrogen bonding with the surface functional groups (Baek et al., 2022; Filice et al., 2022).

In the present work, cationic and anionic dyes were adsorbed differentially on the surface of the Semal-ZnO NPs. The presence of several functional groups like O-H, C=O=C, C=C, and C-H on the surface of the Semal-ZnO NPs, as revealed by FT-IR spectroscopy (Figure 1C), may have played a crucial role in the adsorption of these dyes. The aforementioned functional groups can interact with cationic and anionic dyes through various chemical interactions, such as electrostatic interactions and hydrogen bonding, either alone or in combination. Hence, differential adsorption of MB and MO on Semal-ZnO NPs would depend of different factors, such as the surface charge (point zero charge), different functional groups on the Semal-ZnO NP surfaces, chemical interactions, and nature of the dyes (cationic and anionic) themselves (Khan et al., 2021; Nguyen-Hong et al., 2021).

Furthermore, the Semal-ZnO NPs have a wide bandgap energy (3.11 eV), a high surface area, and crystallinity, which allows them to absorb solar light in the ultraviolet region. Given the narrow molecular sizes of the MB dye particles and the high surface area of Semal-ZnO NPs, there may be more active sites for the adsorption of MB dye molecules than MO. Hence, during adsorption under dark conditions, the dye molecules bind to more of the active sites present on the ZnO NPs. Then, when the ZnO NPs are exposed to solar light, electron–hole pairs are generated by the excitation of electrons from the valence to conduction bands. These photogenerated electron–hole pairs play a crucial role in the photocatalytic degradation process; the specific energy levels of the electrons and holes may also favor the degradation of MB over MO.

It is noted that several other factors, such as intraparticle diffusion, pore diffusion, and operating conditions (e.g., temperature and pH), may also influence the overall adsorption kinetics (Suhaimi et al., 2022; Amdeha, 2023; Gadore et al., 2023; Kanwal et al., 2023; Saharan et al., 2023); therefore, additional experiments may be needed to comprehensively characterize the adsorption kinetics of the degradations of both dyes.

### Application to natural water systems

The efficiencies of the ZnO NPs against MB and MO were investigated in different water samples collected from various natural freshwater sources, namely, FS, PL, AD, BL, and TP, at different time intervals. These water samples were collected periodically from their sources and were pooled and stored separately for further analyses.

**FIGURE 7:**
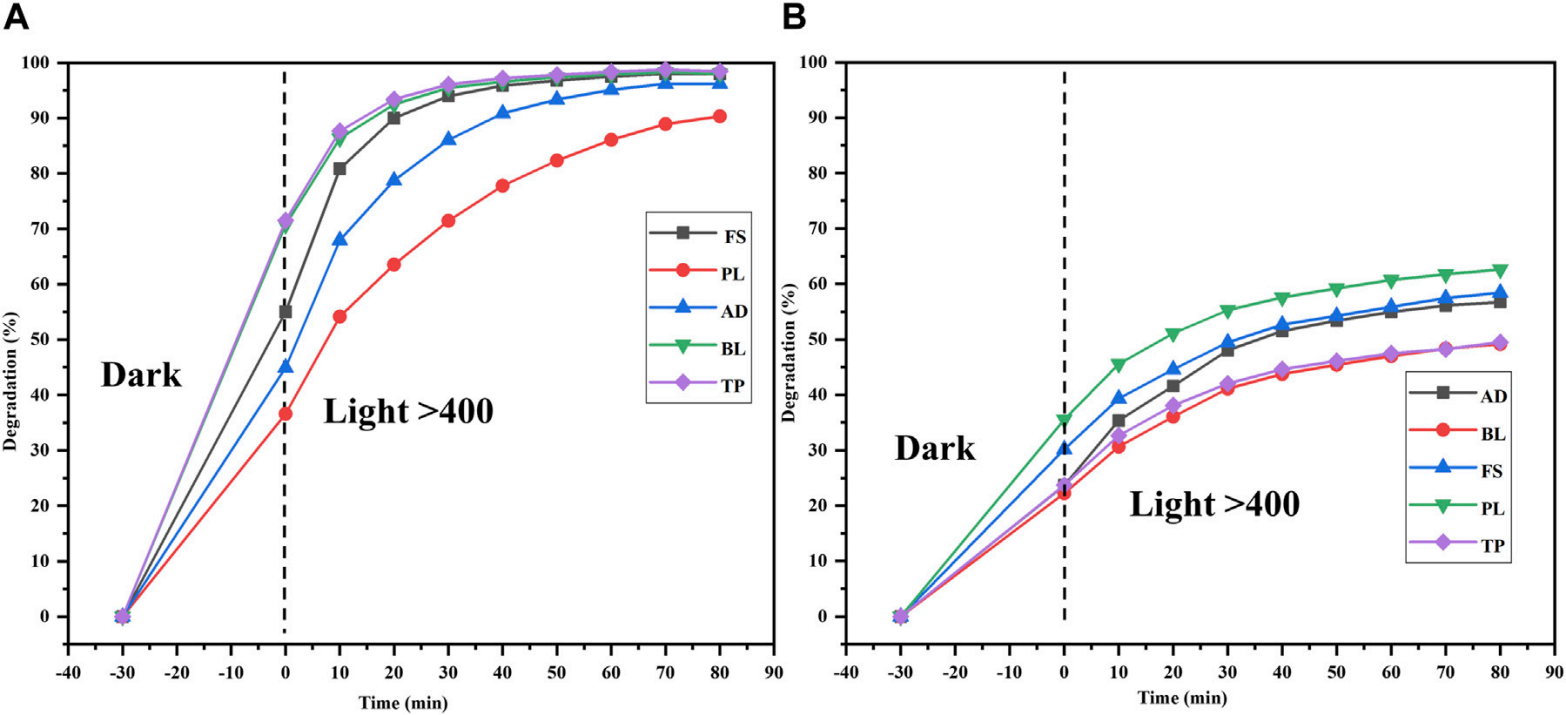
Degradations (%) of the **(A)** MB and **(B)** MO dyes in different water samples (FS, PL, AD, BL, and TP) from the Udaipur region using the biofabricated Semal-ZnO NPs.

To demonstrate catalytic photodegradation, approximately 25 mg of the photocatalyst (Semal-ZnO NPs) was added to a dye solution containing 10 mg/L of MB and MO and different pooled water samples from the natural water sources. The effectiveness of the Semal-ZnO NPs in degrading the dyes was found to be dependent on the water source as well as specific type of dye used. For MB, the rate of photocatalytic degradation was observed to be highest with TP (98.4%) and lowest with PL (90.3%) samples over 90 min of light exposure (Figure 7A). It is worth noting that the rate of photodegradation of MB dye was 99% over 130 min of exposure to sunlight (Figure 4A). Contrarily, the rate of catalytic photodegradation of MO was highest with PL (63.2%) and lowest with TP (49.7%) samples over 90 min of light exposure (Figure 7B).

The presence of competing cationic/anionic species, such as Na^+^, Ca^+^, Fe^3+^, K^+^, Cl^−^, NO_3_^−^, SO_4_^2–^, and HCO_3_^−^, as well as other chemicals in real water samples may account for the slightly higher and lower percentage degradations of MB and MO in different natural water samples (Khan et al., 2022; Ahmad et al., 2023; Gul et al., 2023). Moreover, the specific characteristics of the natural water sources, differential total dissolved solids (TDS), and pH may have influenced the rates of photocatalytic degradation of the cationic and anionic dyes (Fouda et al., 2021; Bhattacharjee et al., 2022; Kumari et al., 2023). The differential photodegradation rates of different dyes in different natural water sources may be correlated with the dissimilar nature and ionic states of the dyes. The rates of photocatalytic degradation in the different water samples used in this study were observed to be TP > BL ≥ FS > AD > PL for the cationic dye MB and PL > FS > AD > BL > TP for the anionic dye MO in the presence of Semal-ZnO NPs as the photocatalyst (Figures 7A,B). Thus, the phytofabricated Semal-ZnO NPs were highly effective and efficient for the degradation of the cationic dye MB in the present pilot experiment.

The present research demonstrates the potential of Semal-ZnO NPs for degrading dyes in natural water samples; however, various other factors, including the nature of the water, its source, and the properties of the dyes, may influence the degradation process significantly in real-world applications.

## Conclusion

In this work, Semal-ZnO NPs were successfully prepared in an environmentally friendly, non-toxic, cost-effective, and sustainable manner using the aqueous leaf extract of *B. ceiba* as the reducing and capping agent as well as synthesis precursor. The formation of the Semal-ZnO NPs was confirmed through its UV-vis spectrum at approximately 390 nm and E_g_ value of 3.11 eV. The morphology and particle size of the Semal-ZnO NPs were evaluated by FT-IR spectroscopy, XRD, FE-SEM with EDS, and HR-TEM; the average grain size was found to be 17 nm. The photodegradation efficiencies of MB and MO dyes were observed to be 99% and 79%, respectively, under solar irradiation when employing the phytofabricated Semal-ZnO NPs as the catalyst. In summary, the results show that the phytofabricated Semal-ZnO NPs are far more effective photocatalysts for the breakdown of MB dye than MO dye in a variety of natural water samples under ambient conditions. To the best of the authors’ knowledge, this is a pioneering report on the utilization of the aqueous leaf extract of the Semal tree in the phytofabrication of Semal-ZnO NPs and their role in water purification. The Semal (*B. ceiba*) tree also offers a high-potential green route for producing Semal-ZnO NPs. This phytofabrication synthesis route using the Semal tree may also pave the path for biosyntheses of other metals, alloys, and their oxide NPs. The Semal-ZnO NPs are expected to have extensive use as powerful agents for environmental remediation and wastewater treatment as they can be robust nano-photocatalysts for the degradation of dyes and potentially other harmful substances in water bodies.

## Data Availability

The original contributions presented in the study are included in the article/Supplementary Material; further inquiries may be directed to the corresponding author.

## Abbreviations

ZnO NPs, zinc oxide nanoparticles; MB, methylene blue (cationic dye); MO, methyl orange (anionic dye); *B*. *ceiba*, *Bombax ceiba* Linn. (Semal); Zn(NO_3_)∙6H_2_O, zinc nitrate hexahydrate.
